# Cardiometabolic Risk Factors Related to Vitamin D and Adiponectin in Obese Children and Adolescents

**DOI:** 10.1155/2013/503270

**Published:** 2013-07-24

**Authors:** Fatih Kardas, Mustafa Kendirci, Selim Kurtoglu

**Affiliations:** ^1^Department of Paediatric Metabolism, Erciyes University, School of Medicine, 38039 Kayseri, Turkey; ^2^Department of Paediatric Endocrinology, Erciyes University, School of Medicine, 38039 Kayseri, Turkey

## Abstract

Obesity-related diseases are becoming the most important causes of mortality worldwide. Several studies have suggested an association between low levels of vitamin D and obesity. In addition, plasma adiponectin levels have been found to be lower in obese subjects. We evaluated the association of metabolic risk factors with both adiponectin and vitamin D levels and that between adiponectin and vitamin D levels. The study consisted of 114 obese and healthy subjects. 25-Hydroxy vitamin D [25(OH)D] levels were positively correlated with adiponectin and HDL-cholesterol (HDL-C) and inversely correlated with body mass index (BMI), LDL-cholesterol (LDL-C), total cholesterol (T-C), triglyceride (TG), fasting glucose, homeostasis model assessment of insulin resistance (HOMA index), systolic blood pressure (SBP), and diastolic blood pressure (DBP). The mean 25(OH)D levels in the obese and nonobese groups were 22.5 ± 5.7 and 32.3 ± 5.8 ng/mL, respectively (*P* < 0.0001). The mean adiponectin level in the obese group was lower than that in the nonobese group (*P* < 0.0001). Lower vitamin D and adiponectin levels were strongly associated with metabolic risk factors and obesity in Turkish children and adolescents.

## 1. Introduction

Obesity is a growing health concern worldwide and is a major cause of morbidity and mortality. Recent studies have suggested that vitamin D deficiency is associated with cardiometabolic risk factors, including obesity, autoimmune diseases, cancer, and insulin resistance [1, 2]. Furthermore, low 25(OH)D levels have been shown to be associated with higher rates of myocardial infarction and diabetes [3–5], and the incidence of hypertension has been found to increase in association with low vitamin D levels [6].

On the basis of current evidence of an inverse association between vitamin D levels and obesity, we performed the first investigation to elucidate the association of obesity with 25(OH)D and adiponectin levels in the Turkish children.

The adiponectin protein is exclusively secreted by adipose tissue into the bloodstream [7] and is abundant in plasma compared to other hormones. Furthermore, low adiponectin levels have been confirmed in patients with diabetes [8], and body fat percentage has been found to be negatively associated with adiponectin levels in adults [7]. Serum adiponectin levels showed an inverse association with hypertension and the homeostasis model assessment of insulin resistance (HOMA index) [9].

However, there are few studies regarding the association of vitamin D and adiponectin levels with cardiometabolic risk factors in obese and healthy children, and to the best of our knowledge, this is the first study to assess these parameters in obese children and adolescents in Turkey.

## 2. Materials and Methods

### 2.1. Participants

We enrolled a total of 114 children and adolescents (age, 10–16 years) who were admitted to the Unit of Paediatric Metabolism of the Child Hospital of Erciyes University Medical Faculty (Kayseri Province, Turkey) from March to May 2011. The study population was divided into 2 groups (obese and nonobese) by body mass index (BMI) (kg/m^2^), which was percentile-specific for gender and age of Turkish children and adolescents [10].

The number of adolescents in the obese (28/63; 44.5%) and nonobese (24/51; 47%) groups was similar.

Healthy, age- and gender-matched subjects were selected from local schools. Obesity was defined as a BMI >90 (kg/m^2^) according to the reference BMI curves for Turkish children [10].

Anthropometric measurements including weight in kilograms (kg), height in centimetres (cm), and BMI for each participant were performed by the same trained nurse using standard devices. Systolic blood pressure (SBP) and diastolic blood pressure (DBP) were measured twice using a mercury sphygmomanometer, after the subject rested for at least 20 min.

### 2.2. Biological Parameters

After overnight fasting, venous blood samples were collected. All samples were obtained during the spring (March to May 2011). Within 3 h of venipuncture, whole blood samples were centrifuged and separated, and serum portions were frozen at −80°C for future adiponectin analysis. Biochemical parameters, such as LDL-cholesterol (LDL-C) (normal range, 100–130 mg/dL), total cholesterol (T-C) (normal range, 160–200 mg/dL), HDL-cholesterol (HDL-C) (normal range, 35–80 mg/dL), triglyceride (TG) (normal range, 40–140 mg/dL), and fasting glucose (normal range, 65–105 mg/dL) levels, were analyzed immediately using standard assay kits (Abbott GmbH & Co. KG, Wiesbaden, Germany). Adiponectin levels were determined using a commercially available enzyme-linked immunosorbent assay (ELISA) kit (BioVendor GmbH, Heidelberg, Germany). Plasma 25(OH)D levels were measured by high-pressure liquid chromatography (HPLC) using ClinRep kits (IRIS Technologies International GmbH, Cursdorf, Germany). Insulin levels were measured using an immunoradiometric assay kit.

The HOMA index to determine insulin resistance was calculated using the formula [fasting insulin (*μ*U/mL) × fasting glucose (mmol/liter)]/22.5 [11]. Vitamin D deficiency was defined as vitamin D levels of <20 ng/mL and vitamin D insufficiency as vitamin D levels of 21–29 ng/mL [12].

### 2.3. Statistical Analysis

Data analysis was performed using SPSS version 17.0 (SPSS. Inc., Chicago, IL, USA). The results were expressed as mean ± SD. The Kolmogorov-Smirnov test was used to determine the normality of the data. Differences between groups were analysed using the Student's *t*-test. Discrete variables were compared using the Pearson *χ* ^2^ test. The Pearson correlation test was used to determine the correlations among the variables.

## 3. Results

The obese group consisted of 63 subjects (32 males, 31 females), and nonobese group consisted of 51 subjects (26 males, 25 females). The mean ages of the study population, obese group, and nonobese group were 13.5 ± 1.6, 13.5 ± 1.7, and 13.4 ± 1.6 years, respectively.

SBP, DBP, T-C, LDL-C, TG, and fasting glucose were higher in the obese group than those in nonobese group (*P* < 0.01 for all variables), whereas 25(OH)D, adiponectin, and HDL-C levels were lower in the obese group (*P* < 0.01 for all variables).

The mean 25(OH)D levels in the overall population, males, and females were 26.9 ± 7.4, 27.2 ± 7.5, and 26.6 ± 7.4 ng/mL, respectively. There was no significant difference in 25(OH)D levels according to gender in the overall population (*P* > 0.05) (Table 1).

All samples were collected during the spring season; therefore, the 25(OH)D reference values were the same for all participants. The descriptive characteristics of the study population (clinical and biological parameters) are shown in Table 2.

**Figure 1:**
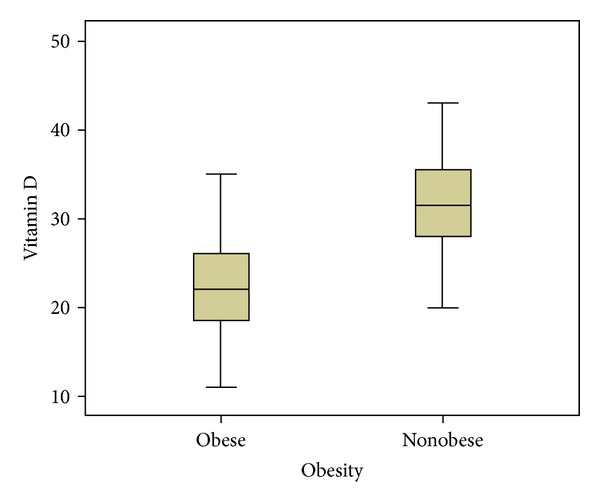
Serum 25(OH)D levels in obese and nonobese subjects.

Association of 25(OH)D levels with obesity is as follows: mean 25(OH)D levels in the total study, obese group, and nonobese group were 26.9 ± 7.4, 22.5 ± 5.7, and 32.3 ± 5.8 ng/mL, respectively. There was a significant difference in 25(OH)D levels between the groups (*P* < 0.0001). Serum 25(OH)D levels are shown in Figure 1.

25(OH)D levels in relation to biochemical and clinical parameters were as follows: 25(OH)D levels showed a positive correlation with adiponectin and HDL-C levels and an inverse correlation with BMI, TG, T-C, LDL-C, fasting glucose levels, HOMA index, SBP, and DBP (Table 3).

**Figure 2:**
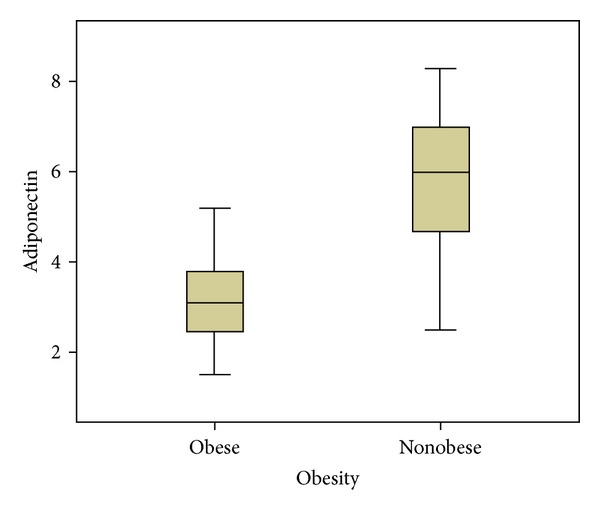
Serum adiponectin levels in obese and nonobese subjects.

The mean adiponectin level in the obese group (3.3 ± 0.89 ng/mL) was lower than that in the nonobese group (6.0 ± 1.4 ng/mL) (*P* < 0.0001) (Table 1). Adiponectin levels according to the groups are shown in Figure 2. BMI, TG, T-C, LDL-C, and fasting glucose levels, HOMA index, SBP, and DBP showed an inverse correlation with adiponectin levels. However, 25(OH)D and HDL-C levels showed a positive correlation with adiponectin levels (Table 4). There was no difference in adiponectin levels according to gender in the total study population (Table 1).

## 4. Discussion

Here we investigated the association of obesity with several metabolic risk factors and both vitamin D and adiponectin levels in children and adolescents. Recent studies suggested that vitamin D levels were lower and vitamin deficiency was more common in obese patients [13, 14]. Furthermore, decreased vitamin D levels in obese patients have been reported due to minimal sun exposure from a sedentary lifestyle, sequestration in fat tissue, and low dietary vitamin D intake because of poor dietary habits [13, 15]. Although we did not discriminate between vitamin D deficient subjects, vitamin D levels were significantly lower in the obese group (*P* < 0.0001). While most findings regarding obesity and vitamin D level come from adult studies, Lenders et al. [16] reported that lower vitamin D level was associated with higher body fat index among obese adolescents.

In this study, we observed an inverse correlation between vitamin D and T-C, LDL-C, and TG levels and a positive correlation between vitamin D and HDL-C levels, which was consistent with previous studies [17–20]. In a study by Gannagé-Yared et al. [19], vitamin D was positively correlated with HDL-C levels in 381 young adults, and a second recent study demonstrated that vitamin D deficiency increased peripheral insulin resistance and thereby altered the lipid profile [21].

Many studies have reported that vitamin D levels were negatively associated with type 1 diabetes and insulin resistance in children and adults [13, 19, 22, 23]. Results of our study demonstrated that lower vitamin D levels were significantly correlated with higher fasting glucose levels and HOMA index (*r* = −0.369, *P* = 0.031 and *r* = −0.480, *P* = 0.035). Furthermore, our study suggested that obesity was a risk factor for lower vitamin D levels, which probably worsened insulin resistance.

Reportedly, the incidence of hypertension increases during the winter months [1, 24], and increased hypertension rates in non-Hispanic blacks with lower vitamin D levels, compared to whites, suggest an association between hypertension and vitamin D levels [25]. Here we found an inverse correlation between vitamin D levels and both SBP and DBP; there was some evidence of a role of vitamin D in hypertension regulation. One is the inhibition of renin gene expression by 1,25(OH)_2_D, which is an active metabolite of 25(OH)D, and also vitamin D inhibits the renin-angiotensin-aldosterone system [26]. Another proposed mechanism involves the direct vascular effects of vitamin D as mediated by the 1*α*-hydroxylase enzyme, in the conversion of 25(OH)D to 1,25(OH)_2_D, which is expressed in vascular smooth muscle cells [27]. Furthermore, Sun and Zemel [28] suggested that the adiponectin gene expression may be upregulated by vitamin D and reported that adipokine synthesis in visceral adipose tissue was regulated by 1,25-hydroxyvitamin D_3_. Tumour necrosis factor alpha affects adiponectin expression, and 1,25-hydroxyvitamin D_3_ regulates tumour necrosis alpha gene [29, 30]. Therefore, we propose that the interaction between vitamin D and adiponectin levels may be an indicator of cardiometabolic risk factors of diseases such as atherosclerosis, because we know that adiponectin protects against atherosclerosis [31]. Nonetheless, further experimental and clinical observations are required to elucidate this interaction. Importantly, we found a positive correlation between 25(OH)D and adiponectin levels in the total cohort of Turkish subjects (*P* < 0.001, *r* = 0.360).

Our results showed that adiponectin levels were lower in obese group (*P* < 0.001) and were inversely correlated (*r* = −0.556, *P* < 0.001) with BMI in accordance with previous reports on adults. However, the association in children and adolescents remains unclear. Adiponectin levels in diabetic patients were found to be lower than those in healthy subjects [7, 8]. In a study reported by Lindsay et al. [32], plasma adiponectin levels were lower in Pima Indians, a group with a high prevalence of obesity and diabetes, whereas a second study demonstrated that adiponectin was strongly associated with insulin sensitivity [33]. Our results showed that plasma adiponectin levels were strongly correlated with fasting glucose levels and HOMA indexes (*r* = −0.224, *P* = 0.016 and *r* = −0.264, *P* = 0.004, resp.), suggesting that hypoadiponectinemia plays a crucial role in insulin resistance and in the development of diabetes. Furthermore, a recent study reported that low serum adiponectin levels were negatively associated with hypertension [9], which confirms our finding of an association between obese and nonobese groups when compared for SBP and DBP (*P* = 0.001, *P* < 0.0001, resp.). Lastly, our study demonstrated a negative correlation between plasma adiponectin levels and both SBP and DBP (*r* = −206, *P* = 0.028 and *r* = −0.394, *P* = 0.000, resp.).

## 5. Conclusion

This is the first study to evaluate the association of multiple metabolic risk factors with both adiponectin and vitamin D levels in Turkish subjects. In this study, we found strong associations between obesity-related parameters, and vitamin D and adiponectin levels in children and adolescents. However, further studies are needed to confirm our findings in larger populations.

## Figures and Tables

**Table 1:** Adiponectin and 25(OH)D levels according to gender.

Parameters	Male (*n* = 58)	Female (*n* = 56)	*P* value
25(OH)D (ng/mL)	27.2 ± 7.5	26.6 ± 7.4	0.662
Adiponectin (*μ*g/mL)	4.58 ± 1.91	4.45 ± 1.64	0.688

**Table 2:** Baseline clinical and biological parameters of study subjects.

Parameters	Total (*n* = 114)	Obese group (*n* = 63)	Nonobese group (*n* = 51)	*P* value^a^
Age (years)	13.5 ± 1.6	13.5 ± 1.6	13.4 ± 1.7	0.835
BMI (kg/m^2^)	24.5 ± 5.4	28.5 ± 2.7	19.6 ± 3.6	<0.0001
SBP (mmHg)	119.7 ± 9.1	122.6 ± 9.6	116.2 ± 7.0	0.001
DBP (mmHg)	66.9 ± 8.9	71.0 ± 8.9	61.9 ± 5.7	<0.0001
HOMA index	1.8 ± 0.55	1.9 ± 0.64	1.6 ± 0.31	0.001
TG (mg/dL)	113.3 ± 25.6	128.7 ± 22.9	94.3 ± 13.1	<0.0001
LDL-C (mg/dL)	94.6 ± 27.5	111.9 ± 24.1	73.3 ± 12.5	<0.0001
T-C (mg/dL)	160.1 ± 27.8	177.6 ± 24.3	138.6 ± 12.4	<0.0001
HDL-C (mg/dL)	44.6 ± 5.9	40.7 ± 3.4	49.4 ± 4.8	<0.0001
25(OH)D (ng/mL)	26.9 ± 7.4	22.5 ± 5.7	32.3 ± 5.8	<0.0001
Adiponectin (*μ*g/mL)	4.5 ± 1.7	3.3 ± 0.89	6.0 ± 1.4	<0.0001
Fasting glucose (mg/dL)	85.2 ± 12.8	89.0 ± 15.0	80.5 ± 7.2	0.003

^a^ *P* values between obese and nonobese groups.

**Table 3:** Correlations of 25(OH)D with other parameters of the total study, obese group, and nonobese group.

Parameters	Obese (*n* = 63)		Nonobese (*n* = 51)		Total population	
	*r*	*P*	*r*	*P*	*r*	*P*
Adiponectin (*μ*g/mL)	−0.147	0.251	−0.23	0.105	0.360	0.000
BMI (kg/m^2^)	−0.199	0.118	−0.073	0.612	−0.553	0.000
TG (mg/dL)	0.199	0.117	0.094	0.513	−0.306	0.001
T-C (mg/dL)	0.167	0.190	−0.35	0.808	−0.360	0.000
HDL-C (mg/dL)	−0.111	0.385	−0.20	0.890	0.404	0.000
LDL-C (mg/dL)	0.179	0.161	0.048	0.736	−0.343	0.000
Fasting glucose (mg/dL)	0.112	0.381	−0.144	0.313	−0.369	0.031
HOMA index	0.075	0.558	−0.147	0.304	−0.480	0.035
SBP (mmHg)	0.33	0.796	0.013	0.926	−0.190	0.043
DBP (mmHg)	−0.66	0.608	−0.252	0.75	−0.392	0.000

**Table 4:** Correlations between adiponectin levels and other parameters in the total study, obese group, and nonobese group.

Parameters	Obese (*n* = 63)		Nonobese (*n* = 51)		Total population	
	*r*	*P*	*r*	*P*	*r*	*P*
25(OH)D (ng/mL)	−0.147	0.251	-0.23	0.105	0.360	0.000
BMI (kg/m^2^)	0.288	0.022	0.095	0.506	−0.556	0.000
TG (mg/dL)	0.143	0.265	0.239	0.091	−0.429	0.000
T-C (mg/dL)	0.110	0.391	0.249	0.078	−0.463	0.000
HDL-C (mg/dL)	−0.145	0.256	−0.013	0.927	0.525	0.000
LDL-C (mg/dL)	0.095	0.459	0.137	0.338	−0.485	0.000
Fasting glucose (mg/dL)	0.110	0.389	−0.041	0.774	−0.224	0.016
HOMA index	0.023	0.861	−0.154	0.282	−0.264	0.004
SBP (mmHg)	0.059	0.645	0.147	0.304	−0.206	0.028
DBP (mmHg)	0.010	0.941	−0.049	0.734	−0.394	0.000
